# Trends in Prevalence of Advanced HIV Disease at Antiretroviral Therapy Enrollment — 10 Countries, 2004–2015

**DOI:** 10.15585/mmwr.mm6621a3

**Published:** 2017-06-02

**Authors:** Andrew F. Auld, Ray W. Shiraishi, Ikwo Oboho, Christine Ross, Moses Bateganya, Valerie Pelletier, Jacob Dee, Kesner Francois, Nirva Duval, Mayer Antoine, Chris Delcher, Gracia Desforges, Mark Griswold, Jean Wysler Domercant, Nadjy Joseph, Varough Deyde, Yrvel Desir, Joelle Deas Van Onacker, Ermane Robin, Helen Chun, Isaac Zulu, Ishani Pathmanathan, E. Kainne Dokubo, Spencer Lloyd, Rituparna Pati, Jonathan Kaplan, Elliot Raizes, Thomas Spira, Kiren Mitruka, Aleny Couto, Eduardo Samo Gudo, Francisco Mbofana, Melissa Briggs, Charity Alfredo, Carla Xavier, Alfredo Vergara, Ndapewa Hamunime, Simon Agolory, Gram Mutandi, Naemi N. Shoopala, Souleymane Sawadogo, Andrew L. Baughman, Adebobola Bashorun, Ibrahim Dalhatu, Mahesh Swaminathan, Dennis Onotu, Solomon Odafe, Oseni Omomo Abiri, Henry H. Debem, Hank Tomlinson, Velephi Okello, Peter Preko, Trong Ao, Caroline Ryan, George Bicego, Peter Ehrenkranz, Harrison Kamiru, Harriet Nuwagaba-Biribonwoha, Gideon Kwesigabo, Angela A. Ramadhani, Kahemele Ng’wangu, Patrick Swai, Mohamed Mfaume, Ramadhani Gongo, Deborah Carpenter, Timothy D. Mastro, Carol Hamilton, Julie Denison, Fred Wabwire-Mangen, Olivier Koole, Kwasi Torpey, Seymour G. Williams, Robert Colebunders, Julius N. Kalamya, Alice Namale, Michelle R. Adler, Bridget Mugisa, Sundeep Gupta, Sharon Tsui, Eric van Praag, Duc B. Nguyen, Sheryl Lyss, Yen Le, Abu S. Abdul-Quader, Nhan T. Do, Modest Mulenga, Sebastian Hachizovu, Owen Mugurungi, Beth A. Tippett Barr, Elizabeth Gonese, Tsitsi Mutasa-Apollo, Shirish Balachandra, Stephanie Behel, Trista Bingham, Duncan Mackellar, David Lowrance, Tedd V. Ellerbrock

**Affiliations:** ^1^Division of Global HIV & TB, Center for Global Health, CDC; ^2^Division of Global HIV & TB, Center for Global Health, CDC Haiti; ^3^Programme National de Lutte contre le VIH/SIDA, Ministry of Health, Haiti; ^4^National Alliance of State & Territorial AIDS Directors, Washington, DC; ^5^National Institute of Health, Mozambique; ^6^Ministry of Health, Mozambique; ^7^Division of Global HIV & TB, Center for Global Health, CDC Mozambique; ^8^Ministry of Health and Social Services, Namibia; ^9^Division of Global HIV & TB, Center for Global Health, CDC Namibia; ^10^Ministry of Health, Nigeria;^11^Division of Global HIV & TB, Center for Global Health, CDC Nigeria; ^12^Ministry of Health, Swaziland; ^13^ITECH, Malawi; ^14^Division of Global HIV & TB, Center for Global Health, CDC Swaziland; ^15^Gates Foundation, Seattle, Washington; ^16^ICAP, New York, New York; ^17^Muhimbili University of Health and Allied Sciences, Tanzania; ^18^National AIDS Control Program, Tanzanian Ministry of Health; ^19^U.S. Department of Defense, Tanzania; ^20^USAID Tanzania; ^21^Division of Global HIV & TB, Center for Global Health, CDC Tanzania; ^22^Global Health, Population and Nutrition, FHI 360, Durham, North Carolina; ^23^Social and Behavioral Health Sciences, FHI 360, Washington, DC; ^24^Infectious Diseases Institute, Makerere University College of Health Sciences, Uganda; ^25^Institute of Tropical Medicine, Department of Clinical Sciences, Belgium; ^26^FHI 360, Zambia; ^27^Division of Global Health Protection, Center for Global Health, CDC, South Africa; ^28^Division of Global HIV & TB, Center for Global Health, CDC Uganda; ^29^Division of Global HIV & TB, Center for Global Health, CDC Zambia; ^30^FHI 360, Tanzania; ^31^Division of Global HIV & TB, Center for Global Health, CDC Vietnam; ^32^Vietnam Authority of HIV/AIDS Control, Vietnam; ^33^Tropical Diseases Research Center, Zambia; ^34^Ministry of Health, Zimbabwe; ^35^Division of Global HIV & TB, Center for Global Health, CDC Zimbabwe.

Monitoring prevalence of advanced human immunodeficiency virus (HIV) disease (i.e., CD4+ T-cell count <200 cells/*μ*L) among persons starting antiretroviral therapy (ART) is important to understand ART program outcomes, inform HIV prevention strategy, and forecast need for adjunctive therapies.*^,^^†^^,^^§^ To assess trends in prevalence of advanced disease at ART initiation in 10 high-burden countries during 2004–2015, records of 694,138 ART enrollees aged ≥15 years from 797 ART facilities were analyzed. Availability of national electronic medical record systems allowed up-to-date evaluation of trends in Haiti (2004–2015), Mozambique (2004–2014), and Namibia (2004–2012), where prevalence of advanced disease at ART initiation declined from 75% to 34% (p<0.001), 73% to 37% (p<0.001), and 80% to 41% (p<0.001), respectively. Significant declines in prevalence of advanced disease during 2004–2011 were observed in Nigeria, Swaziland, Uganda, Vietnam, and Zimbabwe. The encouraging declines in prevalence of advanced disease at ART enrollment are likely due to scale-up of testing and treatment services and ART-eligibility guidelines encouraging earlier ART initiation. However, in 2015, approximately a third of new ART patients still initiated ART with advanced HIV disease. To reduce prevalence of advanced disease at ART initiation, adoption of World Health Organization (WHO)–recommended “treat-all” guidelines and strategies to facilitate earlier HIV testing and treatment are needed to reduce HIV-related mortality and HIV incidence.

Data from 10 countries that requested and received support for ART program evaluations through CDC and agreed to participate in the analysis were included. Three approaches to sampling and analysis were employed (Table 1). In Haiti, Mozambique, and Namibia, where large, centralized, electronic ART patient monitoring systems are employed, all available data from 2004–2015 were analyzed. In each of these countries, 77%–100% of all ART patients and 67%–100% of all ART facilities were captured in the electronic system. In Nigeria, Swaziland, Vietnam, and Zimbabwe, nationally representative samples of ART facilities were selected, with probability of selection proportional to facility size. In Tanzania, Uganda, and Zambia, investigators purposively selected health facilities to represent the range of ART facilities in each country and ensure that the study remained feasible. Among the seven sample-based surveys, a sample frame of study-eligible ART patients was created at each selected facility, and simple random sampling was used to select the sample of records. Eligibility criteria included initiation of ART ≥6 months before data abstraction, during 2004–2015, and at age ≥15 years. Data were abstracted from ART records onto standardized abstraction forms by trained study personnel. Because of variations in the timing of retrospective data collection for the 10 studies (Table 1), the calendar years of ART initiation included in the analysis varied among the countries.

**TABLE 1:** Summary of study designs to assess trends in prevalence of advanced disease at antiretroviral therapy enrollment — 10 countries, 2004–2015

Stage 1: selection of study sites							
Region	Country	Estimated no. ART clinics (yr. of assessment)	Estimated no. adult ART enrollees at ART clinics	No. eligible clinics*	Estimated no. study-eligible adult ART enrollees at eligible clinics	Site sampling technique	No. clinics selected
Southern Africa	Mozambique	379 (2014)	582,000	254	446,379	Census	254
	Namibia	213 (2014)	165,468	213	165,468	Census	213
	Swaziland	31 (2009)	50,767	31	50,767	PPS	16
	Zimbabwe	104 (2008)	103,806	70	93,811	PPS	40
	Zambia	322 (2007)	65,383	129	58,845	Purposive	6
East Africa	Tanzania	210 (2007)	41,920	85	37,728	Purposive	6
	Uganda	286 (2007)	45,946	114	41,351	Purposive	6
West Africa	Nigeria^††^	178 (2009)	168,335	139	167,438	PPS	35
Caribbean	Haiti	200 (2015)	65,000	191	60,705	Census	191
Southeast Asia	Vietnam	173 (2009)	28,090	120	25,000	PPS	30
**Total**	**—**	**2,096**	**1,316,715**	**1,346**	**1,147,492**	**—**	**797**
**Stage 2: selection of study patients**							
**Region**	**Country**	**Age-eligibility criteria (age at ART initiation) (yrs)**	**ART enrollment years covered by analysis**	**Patient sampling technique at selected clinics**	Planned sample size*	**No. eligible medical records** **analyzed**	**Date of data collection**
Southern Africa	Mozambique	≥15	2004–2013	Census	446,379	446,379	Dec 2014
	Namibia	≥15	2004–2012	Census	165,468	165,468	Dec 2013
	Swaziland	≥15	2004–2010	SRS	2,500	2,510	Nov 2011–Feb 2012
	Zimbabwe	≥15	2007–2009	SRS	4,000	3,896^†^	Jan–Jun 2010
	Zambia	≥18	2004–2009	SRS	1,500	1,214^§^	Apr–Jul 2010
East Africa	Tanzania	≥18	2004–2009	SRS	1,500	1,421^¶^	Apr–Jul 2010
	Uganda	≥18	2004–2009	SRS	1,500	1,466**	Apr–Jul 2010
West Africa	Nigeria^††^	≥15	2004–2011	SRS	3,500	3,496	Dec 2012–Aug 2013
Caribbean	Haiti	≥15	2004–2015	Census	60,705	60,705	Jun 2016
Southeast Asia	Vietnam	≥15	2005–2009	SRS	7,587	7,583^§§^	Jan–Jun 2010
**Total**	**—**	**—**	**—**	**—**	**694,639**	**694,138**	**—**

**Abbreviations:** ART = antiretroviral therapy; PPS = probability of selection proportional to size; SRS = simple random sampling.

* To keep sample-based studies feasible, in Zimbabwe, Nigeria, and Vietnam, only facilities with ≥50 adults on ART were eligible for sampling, whereas in Zambia, Uganda, and Tanzania, only facilities that had enrolled ≥300 adults on ART were eligible.

^†^ In Zimbabwe, 23 of 3,919 selected patients with either missing gender (n = 12), or missing outcome (n = 11) were excluded from analysis.

^§^ In Zambia, among 1,457 records sampled, 243 were excluded because of noncompliance with simple random sampling procedures at one site.

^¶^ In Tanzania, among 1,458 records sampled, one patient was excluded because of absence of age data at ART initiation, and 36 patients enrolled in 2004 were excluded because of small sample size for 2004.

** In Uganda, among 1,472 records sampled, six patients were excluded because of absence of age data at ART initiation.

^††^ In Nigeria, implicit stratification was used in the sampling approach.

^¶¶^ In Vietnam, among 7,587 records sampled, four observations were excluded because information on gender was missing.

The CD4+ T-cell count (CD4) measured in the 6 months before ART initiation and closest to the date of ART initiation was considered the baseline CD4. For each of the 10 countries and for each calendar year, the percentages of adult patients with baseline CD4 <100, <200, and <350 cells/*μ*L are described with percentages and 95% confidence intervals accounting for survey design. Bivariate logistic regression models accounting for survey design were used to evaluate statistical significance of changes in percentages over calendar years, with the likelihood ratio test used to assess departure from linear trend over time. Trends in median baseline CD4 at ART initiation over time are described, and a linear regression model, accounting for survey design, was used to assess statistical significance of changes.

Across the 10 countries, 694,138 adult ART patient records were analyzed from 797 ART facilities (Table 1). The overall percentage of new ART enrollees during 2004–2015 with missing baseline CD4 ranged from 9% in Swaziland to 53% in Zimbabwe. In the three countries providing more recent national electronic medical record data, prevalence of advanced disease at ART initiation declined from 73% to 37% during 2004–2014 in Mozambique, from 80% to 41% during 2004–2012 in Namibia, and from 75% to 34% during 2004–2015 in Haiti (Table 2) (supplemental figure; https://stacks.cdc.gov/view/cdc/45821). In addition, over the same periods, prevalence of CD4 <100/*μ*L declined from 39% to 18% in Mozambique, from 39% to 16% in Namibia, and from 49% to 20% in Haiti. Prevalence of CD4 <350/*μ*L at ART initiation also declined over time in all three countries. Over the same periods, significant increases in median CD4 count at ART initiation were observed in Mozambique (from 128/*μ*L to 261/*μ*L; p<0.001), in Namibia (from 125/*μ*L to 230/*μ*L; p<0.001), and in Haiti (from 103/*μ*L to 297/*μ*L; p<0.001) (Figure).

**TABLE 2:** CD4 distribution among adult antiretroviral therapy enrollees, by calendar year of ART initiation — 10 countries, 2004–2015

Country	CD4 distribution	Overall			Year (%)												p-value*
		No.	Total No.	% (95% CI)	2004	2005	2006	2007	2008	2009	2010	2011	2012	2013	2014	2015	
Southern Africa Mozambique	CD4<100	66,183	282,129	23 (23–24)	39	39	34	31	29	29	28	25	21	18	18	—	<0.001
	CD4<200	138,453	282,129	49 (49–49)	73	71	68	65	63	62	58	53	44	38	37	—	<0.001
	CD4<350	233,344	282,129	83 (83–83)	93	90	91	91	90	91	90	89	85	73	71	—	<0.001
	Missing	164,250	446,379	37	42	33	31	30	31	32	32	30	32	39	50	—	—
Namibia	CD4<100	16,724	82,774	20 (20–20)	39	35	29	26	26	22	17	13	16	—	—	—	<0.001
	CD4<200	48,555	82,774	59 (58–59)	80	76	76	75	76	71	58	36	41	—	—	—	<0.001
	CD4<350	77,351	82,774	93 (93–94)	97	95	95	95	95	95	95	91	91	—	—	—	<0.001
	Missing	82,694	165,468	50	72	78	79	76	42	38	37	34	30	—	—	—	—
Swaziland	CD4<100	770	2,296	34 (31–36)	32	50	44	41	39	36	24	—	—	—	—	—	0.035
	CD4<200	1,550	2,296	67 (63–71)	72	87	86	78	77	69	54	—	—	—	—	—	0.028
	CD4<350	2,168	2,296	95 (93–96)	90	98	98	95	97	95	92	—	—	—	—	—	0.592
	Missing	214	2,510	9	33	15	18	5	8	10	5	—	—	—	—	—	—
Zambia	CD4<100	310	849	36 (33–40)	23	36	37	38	33	35	—	—	—	—	—	—	0.792
	CD4<200	601	849	70 (67–74)	77	73	76	67	69	65	—	—	—	—	—	—	0.287
	CD4<350	810	849	96 (94–97)	100	93	95	96	96	95	—	—	—	—	—	—	0.562
	Missing	365	1,214	30	73	57	32	23	16	16	—	—	—	—	—	—	—
Zimbabwe	CD4<100	757	1,820	42 (39–45)	—	—	—	46	40	40	—	—	—	—	—	—	0.092
	CD4<200	1,424	1,820	78 (74–81)	—	—	—	84	75%	75	—	—	—	—	—	—	0.042
	CD4<350	1,767	1,820	97 (95–98)	—	—	—	97	97%	97	—	—	—	—	—	—	0.756
	Missing	2,076	3,896	53	—	—	—	55	50%	55	—	—	—	—	—	—	—
East Africa Tanzania	CD4<100	432	1,085	41 (37–44)	—	40	50	40	41%	37	—	—	—	—	—	—	0.581
	CD4<200	804	1,085	77 (74–80)	—	77	80	79	77%	77	—	—	—	—	—	—	0.994
	CD4<350	1039	1,085	97 (95–98)	—	94	99	97	95%	99	—	—	—	—	—	—	0.132
	Missing	336	1,421	24	—	24	28	22	23%	22	—	—	—	—	—	—	—
Uganda	CD4<100	438	1,166	36 (33–39)	54	50	49	30	30	28	—	—	—	—	—	—	<0.001^†^
	CD4<200	859	1,166	74 (71–76)	89	85	83	78	67	60	—	—	—	—	—	—	<0.001^†^
	CD4<350	1,127	1,166	96 (95–97)	99	96	99	95	99	95	—	—	—	—	—	—	0.122
	Missing	300	1,466	20	31	27	23	20	17	16	—	—	—	—	—	—	—
West Africa Nigeria	CD4<100	884	2,876	31 (27–34)	9	36	40	31	31	32	29	25	—	—	—	—	0.001^§^
	CD4<200	1,792	2,876	63 (59–67)	68	67	77	65	63	66	60	53	—	—	—	—	0.043^§^
	CD4<350	2,666	2,876	93 (91–94)	96	91	94	92	94	93	92	92	—	—	—	—	0.576
	Missing	620	3,496	18	33	21	20	21	18	13	16	20	—	—	—	—	—
Caribbean Haiti	CD4<100	10,835	47,209	23 (23–23)	49	51	42	32	26	21	23	22	21	19	18	20	<0.001
	CD4<200	20,578	47,209	44 (43–44)	75	79	77	68	55	46	46	42	39	36	32	34	<0.001
	CD4<350	35,306	47,209	75 (74–75)	92	94	94	90	84	86	83	76	72	70	60	59	<0.001
	Missing	25,837	60,705	43	24	36	37	33	37	30	35	31	31	32	33	53	—
Southeast Asia Vietnam	CD4<100	3,015	5,228	58 (55–60)	—	74	63	58	59	52	—	—	—	—	—	—	0.007
	CD4<200	4,384	5,228	84 (81–86)	—	91	87	84	86	80	—	—	—	—	—	—	0.046
	CD4<350	5,038	5,228	96 (95–97)	—	98	97	95	97	96	—	—	—	—	—	—	0.533
	Missing	2,355	7,583	31	—	39	42	36	28	25	—	—	—	—	—	—	—

**Abbreviations:** ART = antiretroviral therapy; CD4 = CD4+ T-cell count (cells/*μ*L); CI = confidence interval.

* P-value derived from logistic regression, accounting for survey design, comparing the percentage of enrollees below the specified CD4 threshold in the most recent calendar year with the corresponding percentage in the earliest calendar year with data available.

^†^ Specified p-values were derived from logistic regression including calendar year of ART initiation as a continuous variable because of observed linear trend.

^§^ In Nigeria, the p-value compares percentages in 2006 with percentages in 2011, to best capture recent declines in prevalence of advanced disease.

**FIGURE:**
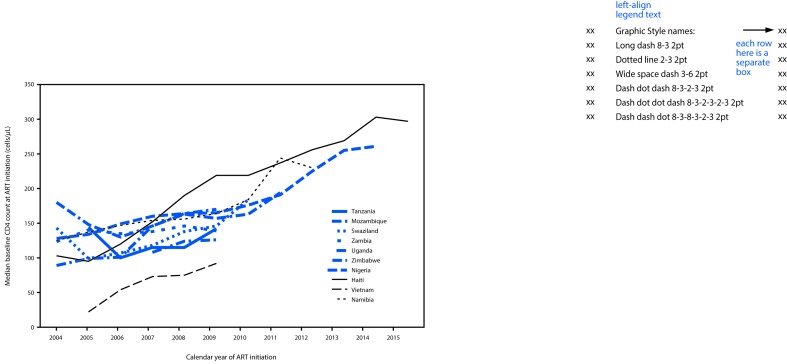
Trends in median CD4+ T-cell count at antiretroviral therapy (ART) initiation — 10 countries, 2004–2015

In the seven countries with less recent data, statistically significant declines in prevalence of advanced disease were observed in five countries (Table 2). Prevalence of advanced disease at ART initiation declined from 72% to 54% in Swaziland (2004–2010), from 84% to 75% in Zimbabwe (2007–2009), from 89% to 60% in Uganda (2004–2009), from 68% to 53% in Nigeria (2004–2011), and from 91% to 80% in Vietnam (2005–2009) (Table 2) (supplemental figure; https://stacks.cdc.gov/view/cdc/45821). Over the same periods in Swaziland, Uganda, and Vietnam, statistically significant increases in median baseline CD4 from 143/*μ*L to 184/*μ*L (p<0.001), 89/*μ*L to 170/*μ*L (p<0.001), and 22/*μ*L to 92/*μ*L (p = 0.014), respectively, were observed (Figure).

## Discussion

This analysis of 694,138 medical records from 10 low- and middle-income countries (LMIC), contributes several findings relevant for ART programs in resource-limited settings. Observed declines in the prevalence of advanced disease at ART initiation in eight countries and increases in median baseline CD4 at ART initiation in six countries are likely due to increasing access to HIV testing and treatment (e.g., increasing numbers of facilities providing testing and treatment services), and increasingly inclusive ART eligibility guidelines (*1*). Despite this encouraging progress, however, a significant percentage of ART enrollees still started ART with advanced disease in recent years. In Haiti, which provided the most recent data on ART enrollees for this analysis (2015), and which historically has had higher than average median CD4 at ART initiation compared with other LMIC (Table 2) (*2*,*3*), the percentage of ART enrollees with CD4 <200/*μ*L was 34% in 2015. Similarly, in Mozambique in 2014, 37% of patients started ART with advanced disease. Although recent data from the 10 countries are limited, these data and data from a recent meta-analysis, which reported mean CD4 count at ART initiation for 27 LMIC in 2011–2013 of 186 cells/*μ*L (*3*), suggest at least a third of ART patients in LMIC initiated ART with advanced disease in 2015. To reduce prevalence of advanced disease at ART initiation in LMIC, continued attention to programmatic strategies facilitating earlier HIV testing and linkage to care are needed, in addition to adoption of WHO-recommended universal ART eligibility (“treat-all”) guidelines for persons living with HIV (*3*), which stipulate that all patients become eligible for ART on the day of HIV diagnosis, regardless of CD4 count at HIV diagnosis. Early ART for all persons living with HIV could improve ART program outcomes and HIV prevention impact (*4*,*5*). For example, in the Strategic Timing of Antiretroviral Therapy (START) trial, initiating ART for patients with CD4 >500/*μ*L rather than deferring ART initiation until more advanced disease stages, was shown to reduce risk for a composite endpoint of any serious acquired immunodeficiency syndrome (AIDS)–related event, non-AIDS–related event, or death by 57% (*5*). In addition, early rather than deferred ART for HIV-positive persons in a serodiscordant relationship was found to reduce HIV transmission to the HIV-negative partner by approximately 96% (*4*). Among the 10 countries studied, “treat-all” guidelines have been adopted nationwide in nine (Haiti, Mozambique, Namibia, Nigeria, Swaziland, Tanzania, Uganda, Zambia, and Zimbabwe), whereas Vietnam is beginning to phase in “treat-all” guidelines with nationwide adoption planned by 2020.

Given the low median baseline CD4 from Vietnam in 2009 (92/*μ*L), much lower than Haiti’s median baseline CD4 the same year (219/*μ*L), evaluation of more recent trends in baseline CD4 is warranted. With Vietnam’s epidemic largely involving men who inject drugs, late presentation for ART might be partly explained by suboptimal health-seeking behavior in this population (*6*). In Vietnam and similar LMIC, continued monitoring of the prevalence of advanced HIV disease at ART initiation is necessary to inform understanding of ART program access, outcomes, and prevention strategies (because baseline CD4 gives an indication of how long ART enrollees have lived with an unsuppressed viral load). Comparing prevalence of advanced disease at ART initiation among demographic groups (e.g., nonpregnant females, pregnant females, and males) or among more affected population groups (e.g., sex workers and persons who inject drugs) can inform which populations are being reached late and therefore require targeted interventions (*1*).

Recent WHO guidelines recommend a differentiated approach to treatment of persons living with HIV.^¶^ This approach means that patients initiating ART with advanced HIV disease require additional specialized care to ensure optimal outcomes. For example, tuberculosis (TB) is common among patients starting ART with advanced HIV disease, and remains the most common cause of death, accounting for approximately 40% of deaths, half of which are undiagnosed before death (*7*). Based on recent evidence from a randomized trial (*8*), WHO recommends that the lateral flow urine lipoarabinomannan assay may be used to assist in the rapid diagnosis and treatment of disseminated TB among persons living with HIV admitted to hospital with CD4 <100/*μ*L and symptoms of TB. WHO conditionally recommends the same screening approach for adult outpatients. Early identification and treatment of disseminated TB can reduce all-cause mortality (*8*). In addition, plasma screening for cryptococcal antigen (CrAg) among patients with CD4 <100/*μ*L and consideration of preemptive treatment with fluconazole for CrAg-positive patients might reduce 12-month ART mortality (*9*). Co-trimoxazole prophylaxis for ART enrollees with CD4 <350/*μ*L has been shown to reduce mortality (*10*). Use of these adjunctive therapies could help reduce relatively high 12-month mortality among people taking ART in LMIC (*1*).

Given the importance of baseline CD4 in determining eligibility for adjunctive therapies that have the potential to reduce mortality, it is concerning that 40% of the 694,138 medical records reviewed lacked documentation of the baseline CD4, with country-specific rates ranging from 9% in Swaziland to 53% in Zimbabwe. Quality improvement measures to ensure availability of baseline CD4 data for clinical decision-making are warranted.

The findings in this report are subject to at least three limitations. First, cohort data varied in size and generalizability; statistical significance of trends in baseline CD4 over time is more likely with larger sample sizes and more calendar years of available data. Second, missing data on CD4 at ART initiation might have introduced measurement error for summary estimates. Third, in several countries, data on more recent ART enrollees are needed to inform estimates of the current prevalence of advanced HIV disease at ART initiation.

Encouraging reductions in the prevalence of advanced disease at ART initiation were observed in eight of the 10 countries studied. This reflects the rapid scale-up of HIV testing and treatment services in LMIC since 2004 and evolution of HIV treatment guidelines encouraging earlier ART initiation. However, an estimated one third of new ART enrollees in LMIC in 2015 started ART with advanced disease, indicating that continued scale-up of interventions to facilitate earlier testing and treatment are needed. For those ART enrollees who do initiate ART late (*3*), ensuring availability of WHO-recommended adjunctive therapies could help reduce morbidity and mortality during ART.

**Summary**

**What is already known about this topic?**

Monitoring prevalence of advanced human immunodeficiency virus (HIV) disease (i.e., CD4+ T-cell count <200 cells/*µ*L) among persons initiating antiretroviral therapy (ART) is important to help understand ART program outcomes, inform HIV prevention strategies, and forecast need for adjunctive therapies.

**What is added by this report?**

In an analysis of 694,138 adult ART records from 10 countries, the prevalence of advanced disease at ART initiation during 2004–2015 declined in eight countries. In Mozambique (2004–2014), Namibia (2004–2012), and Haiti (2004–2015), prevalence of advanced disease at ART initiation declined from 73% to 37% (p<0.001), 80% to 41% (p<0.001), and 75% to 34% (p<0.001), respectively. In the remaining seven countries with data available for 2004–2011, significant declines in prevalence of advanced disease were observed in Nigeria, Swaziland, Uganda, Vietnam, and Zimbabwe.

**What are the implications for public health practice?**

Declines in the prevalence of advanced disease at ART enrollment over time in most countries are encouraging, but in 2015, approximately a third of new ART patients still initiated ART late. Adoption of World Health Organization–recommended “treat-all” guidelines and strategies to facilitate earlier HIV testing, and treatment are needed. These strategies would help reduce HIV-related mortality and HIV incidence.

## Footnotes

* World Health Organization. Guidelines on co-trimoxazole prophylaxis for HIV-related infections among children, adolescents and adults. http://www.who.int/hiv/pub/guidelines/ctx/en/.

† World Health Organization. The use of lateral flow urine lipoarabinomannan assay (LF-LAM) for the diagnosis and screening of active tuberculosis in people living with HIV. http://apps.who.int/iris/bitstream/10665/193633/1/9789241509633_eng.pdf?ua=1&ua=1.

§ World Health Organization. Rapid advice: diagnosis, prevention and management of cryptococcal disease in HIV-infected adults, adolescents and children. http://apps.who.int/iris/bitstream/10665/44786/1/9789241502979_eng.pdf.

¶ World Health Organization. Guideline on when to start antiretroviral therapy and on preexposure prophylaxis for HIV. http://www.who.int/hiv/pub/guidelines/earlyrelease-arv/en/.
